# HMGB1/IL-1β complexes in plasma microvesicles modulate immune responses to burn injury

**DOI:** 10.1371/journal.pone.0195335

**Published:** 2018-03-30

**Authors:** Leon G. Coleman, Robert Maile, Samuel W. Jones, Bruce A. Cairns, Fulton T. Crews

**Affiliations:** 1 Department of Pharmacology, School of Medicine, University of North Carolina at Chapel Hill, Chapel Hill, NC, United States of America; 2 Bowles Center for Alcohol Studies, School of Medicine, University of North Carolina at Chapel Hill, Chapel Hill, NC, United States of America; 3 North Carolina Jaycee Burn Center, Department of Surgery, University of North Carolina at Chapel Hill, Chapel Hill, NC, United States of America; 4 Department of Microbiology and Immunology, University of North Carolina at Chapel Hill, Chapel Hill, NC, United States of America; Universita degli Studi di Torino, ITALY

## Abstract

Modulating immune responses to sepsis and trauma remain one of the most difficult challenges in modern medicine. Large burn injuries (LBI) are a severe form of trauma associated with sepsis, immune impairment, and mortality. Immune dysfunction after LBI is complex, involving both enhanced and impaired immune activation. The release of Damage-Associated Molecular Patterns (DAMPs), such as HMGB1, and cytokines (e.g. IL-1β) creates an environment of immune dysfunction often leading to end organ failure and death. Both HMGB1 and IL-1β have been found to play critical roles in sepsis and post-burn immune dysfunction. HMGB1 and IL-1β have been shown previously to form potent complexes *in vitro*. We recently identified the presence of HMGB1/IL-1β heterocomplexes in human tissue. We now find HMGB1/IL-1β complexes in human and mouse plasma, and identify a synergistic role of HMGB1/IL-1β complexes in post-burn immune dysfunction. In both humans and mice, we found that HMGB1 was enriched in plasma microvesicles (MVs) after LBI. HMGB1 was found form complexes with IL-1β. Using flow cytometry of mouse plasma MVs, we identified an increase in an HMGB1+/IL-1β+ MVs. Using co-IP, HMGB1 was found to bind the pro-form of IL-1β in mouse and human plasma. Pro-IL-1β, which is traditionally considered inactive, became active when complexed with HMGB1. Human THP-1 monocytes treated with HMGB1-pro-IL-1β complexes showed increased transcription of LBI associated cytokines IL-6 and IFNβ along with suppression of iNOS, mimicking findings associated with LBI. These findings identify that HMGB1/IL-1β complexes released after burn injuries can modulate immune responses, and microvesicles are identified as a novel reservoir for these immune mediators. These complexes might serve as novel immune targets for the treatment of systemic immune responses due to LBI or other causes of sepsis.

## Introduction

Managing systemic responses to sepsis and trauma remains one of the most elusive challenges in modern medicine. Immune dysregulation is suspected as an underlying cause of organ dysfunction and failure. Both Damage-Associated Molecular Patterns (DAMPs) and cytokines are involved immune dysfunction after burns, trauma and sepsis [1–3]; however, attempts at individual cytokine-directed therapies in humans have been unsuccessful [4, 5]. Large burn injuries (LBI) represent one of the most severe forms of trauma, with large burns resulting in profound innate immune dysfunction. This involves an acute systemic inflammatory response phase, following by persistent immune dysregulation [6]. The LBI-associated initial immune response is a severe form of sepsis that includes systemic release of several cytokines and chemokines. Multiple studies have described the cytokine responses after large burn injuries [7–10]. However, the shear enormity of the immune responses associated with burn injuries and sepsis, as well as the involvement of multiple immune molecules makes the development targeted immune therapies difficult. In addition to cytokines, DAMPs regulate immune responses after injury through their interaction with pattern-recognition receptors such as Toll-like Receptors (TLRs). The interactions of cytokines and DAMPs is likely critical for the regulation of innate immune function. DAMPs such as HMGB1, and the cytokine IL-1β have been found to interact directly, forming potent heterocomplexes *in vitro* [11–13]. Therefore, it is necessary to understand the interaction between underlying modulators of these innate immune signals, in order to dissect these complex immune responses.

Both the DAMP High-Motility Group Box-1 (HMGB1) and the cytokine IL-1β have been found to play critical roles in sepsis and post-burn immune dysfunction [3, 7, 14]. HMGB1 is a nuclear histone binding protein that upon its release acts a DAMP, and has been identified as a critical mediator of mortality in peritoneal and endotoxin-induced sepsis [1, 3]. Passive HMGB1 release occurs after tissue injury, necrotic cell death or cellular stress. In the case of immune cells, however, HMGB1 is actively secreted following activation. Active secretion of HMGB1 involves its packaging and release in microvesicles (MVs) [15–17]. MVs are a class of extracellular vesicles released by cells during various physiological or pathological processes [18–20]. Previously considered mere cellular “dust” or debris, MVs and other extracellular vesicles are now recognized as critical mediators of intracellular signaling [21, 22]. MVs are a fundamental mechanism of cell-cell communication. MVs carry cytokines, DAMPs, and miRNA and are able to deliver their cargo to recipient cells. Therefore, MVs can regulate immune and other signaling pathways of target cells in a variety of disease states such as cancer, renal injury, and sepsis [21, 23, 24]. In this report we have focused on MVs, rather than exosomes or larger apoptotic bodies since we have focused on HMGB1 and IL-1β signaling after burn injury.

Prior studies find that both HMGB1 and IL-1β can be secreted in MVs, utilizing unconventional secretion mechanisms in certain settings [25, 26]. Following LBI, HMGB1 is secreted in plasma up to three weeks after the initial injury, and correlates with onset of sepsis and mortality [14]. Secreted HMGB1 can regulate immune responses in target cells through multiple mechanisms. HMGB1 directly binds the Toll-like Receptor 4 (TLR4) or RAGE leading to NFκB activation and maintenance of innate immune responses. However, HMGB1 can also act as an immune chaperone, forming complexes with other immune molecules such as IL-1β, miRNA let-7, endotoxin, CXCL12 or peptidoglycans to modulate their activities through their own receptors, such as the IL-1β receptor [11, 12, 17, 27]. These complexes are either more potent or, in the case of certain nucleic acids, require HMGB1 for their TLR-mediated signaling [12, 28]. We recently found the presence of HMGB1/cleaved-IL-1β complexes *in vivo* and in human brain tissue [29]. Here, we hypothesized that HMGB1/IL-1β complexes would play an important role in post-burn immune responses. Since HMGB1 and IL-1β are released in MVs, we hypothesized that MVs would carry these immunogenic heterocomplexes.

We assessed HMGB1 and IL-1β in plasma MVs using a mouse model of LBI, as well as in plasma from human burn patients. During the acute and subacute phases after burn injury we found that HMGB1 and IL-1β were increased acutely in plasma MVs (≤48), but not MV-depleted plasma, persisting into the subacute phase (72-120h). Flow cytometry analysis of plasma MVs showed an increase in MVs that contain both HMGB1 and IL-1β up to 72 hours after injury. Co-immunoprecipitation found that HMGB1 formed complexes with IL-1β in both human and mouse plasma. Traditionally, IL-1β signaling is thought require cleavage of pro-IL-1β by its activating protease caspase-1 with subsequent vesicular or free release [30–33]. However, we observed that HMGB1 formed complexes with pro-IL-1β in plasma. When coupled with HMGB1, pro-IL-1β was active, causing innate immune genes induction in human THP-1 monocytes, in a similar manner to cleaved-IL-1β. Immune gene induction patterns were similar to those seen following burn injury, specifically the enhancement of IL-6 and IFNβ gene transcription coupled with the suppression of iNOS [34]. Thus, the sustained release of HMGB1 and IL-1β complexes in MVs may contribute to the ongoing immune dysfunction seen with burn injuries, and could represent a novel immune target.

## Materials and methods

### Reagents

The following reagents were purchased from Sigma-Aldrich (St Louis, USA): HMGB1 ELISA kit was purchased from IBL International (Hamburg, Germany). Primary antibodies from Abcam: ChIP grade rabbit polyclonal anti-HMGB1 (ab18256), goat polyclonal anti-β actin (ab8229). Primary antibodies from R&D Systems: goat polyclonal anti-IL-1β for Western Blot (AF-401-NA), rat monoclonal anti-mouse IL-1β for flow cytometry. Secondary antibodies for western blot were purchased from Rockland: donkey anti-rabbit DyLight™ 680 (611-744-127) and donkey anti-goat DyLight™ 800 (605-745-125). Antibodies from Biolegend: PE-HMGB1 (651404), IgG 2b PE-isotype control (400313). Antibodies from Life Technologies: AF488 goat anti-rat secondary (A11006). Complete protease inhibitor tablets were purchased from Roche.

### Human burn patient plasma

Adult patients admitted to the North Carolina Jaycee Burn Center at the University of North Carolina at Chapel Hill hospital were enrolled into the study after written obtained consent was obtained. The University of North Carolina at Chapel Hill IRB and the Office of Human Research Ethics approved this specific study to collect blood samples from patients and healthy volunteers (UNC#04–1437). We obtained informed written and oral informed consent from the patient or Legally Authorized Representative (LAR). Minors were included in this study and informed consent obtained from the patient’s parent or LAR. Subsequent assent was obtained from children who were minors (age 7–14 years) or adolescents (age 15–17 years) using consent forms. The University of North Carolina at Chapel Hill IRB and the Office of Human Research Ethics determined that assent of children younger than 7 years of age was be waived. Where the potential subject was younger than 18 years of age, consent was obtained from the patient’s parent or legal guardian. Subsequent assent was obtained from children who were minors (age 7–14 years) or adolescents (age 15–17 years) using consent forms. The UNC Biomedical IRB determined that assent of children younger than 7 years of age was be waived and informed consent was given by parents or LAR. Consent forms were subjected to review and approval by the Institutional Review Board of the University of North Carolina at Chapel Hill School of Medicine (Protocol #04–1437). Enrollment criteria of patients consisted of: consented patients of all ages admitted to the North Carolina Jaycee Burn Center with burn of any severity, TBSA ≥ 0.5%. There were no exclusions based on race, gender, pregnancy, immune status or ethnicity. Three samples of blood were obtained from each patient enrolled in the study. Two 8.5 ml Vacutainer glass whole-blood tubes containing 1.5 ml of ACD solution A (trisodium citrate, 22.0g / L; citric acid, 8.0 g / L; dextrose 24.5 g / L) were filled with whole blood at each sample collection (Becton Dickinson, Franklin Lakes, NJ). The first blood sample was obtained within the first 24 hours following initial burn injury early during admission. The second was obtained at 24–48 hours following injury, and the third was obtained at 48–72 hours following injury. At each time point, a complete blood count with differential and basic metabolic panel was obtained as well. Clinical data including age, sex, burn degree, length of stay, and burn size were documented throughout each patient’s stay in the burn intensive care unit. Patients with burns greater than or equal to 15% total body surface area (TBSA) were considered to have large burn injury (LBI), patients with burns of less than 15% TBSA were considered to have small burns. Blood samples were similarly collected from 10 healthy volunteers at one time point. Serum IL-1β concentrations were measured using Quantikine Human IL-1 beta Immunoassay ELISA (R&D Systems, MN, USA)

### Mouse 20% total body surface area (TBSA) model

All protocols were performed in strict accordance with the Guide for the Care and Use of Laboratory Animals of the National Institute of Health. The study protocol was approved by the University of North Carolina Institutional Animal Care and Use Committee (#15–192.0) with ethically appropriate experimental design; all animals were housed in American Association for Accreditation of Laboratory Animal Care (AAALAC; an international private, nonprofit organization that promotes the humane treatment of animals in science through voluntary accreditation and assessment programs) accredited facilities with full time veterinarians on staff. Close observation of animals was performed at all times. All appropriate measures were followed to alleviate suffering. All thermal injuries and non-survival surgeries were performed under isoflurane and pentobarbital anesthesia respectively. Mice underwent a 20%TBSA thermal injury as described previously [35]. Briefly female, C57B/6 mice (6–8 weeks old, 15-20g) are anesthetized with isoflurane and shaved before undergoing four ten second exposures with a copper rod heated to 100°C in a water bath, resulting in a full-thickness 20% total body surface area burn. Mice were resuscitated with Ringer’s lactate solution (0.1mL/g body weight) and received buprenorphine (2mg/kg, subcutaneous) for pain control. Ongoing pain control was provided ad lib through morphine sulfate-supplemented water (60μg/20g mouse) throughout the experimental period. Sham treated mice received the same treatment and pain medication without heated copper rod application. This protocol is associated with a very low mortality rate of approximately 1%. Mice were monitored at least twice a day for the first 48–72 hrs post procedure or until they are stabilized. Once the mice were stable, they were monitored every other day. If mice developed overt symptoms of trauma and if not easily treated for their illness (hunched, dehydrated, struggling with breathing, lost >15 percent body weight, were inactive or suffered lesions) they were euthanized immediately using inhaled isoflurance (Drop Method), followed by cervical dislocation. There were never any unexpected deaths. Mice were sacrificed by transcardial perfusion as described previously [36–38]. Briefly, mice were anesthetized with sodium pentobarbital (100mg/kg, i.p.). Mice were then sacrificed by transcardiac perfusion with 0.1M PBS.

### Microvesicle isolation

MVs are isolated by sequential centrifugation as described previously [39]. Briefly, media was centrifuged at 2000g for 20 minutes to remove cells. Supernatant was then centrifuged at 10,000 for 30 minutes to remove cellular debris. Remaining supernatant was then centrifuged at 21,000g for 1 hour. The MV-containing pellet was washed in PBS and centrifuged again at 21,000g. The MV pellet was suspended in 50μl appropriate buffer for analysis. This preparation results in MVs ranging between 100 nm and 1 μm in diameter.

### ELISA measurements of plasma HMGB1 and IL-1β

HMGB1 levels were determined in plasma or MVs by ELISA (IBL, Germany) according to the manufacturer’s instruction with modification. Plasma samples were diluted 1:25 in Tris lysis buffer containing 7.4% EDTA, 3.8% EGTA and 1% Triton X-100. Samples were then treated with perchloric acid (PCA, BioVision catalog #K808) to liberate HMGB1 from its binding partners to allow for measurement of total HMGB1 as previously described [40]. MV samples were added directly to ELISA after PCA treatment without prior dilution. The purified HMGB1-containing supernatant was then assessed by ELISA. IL-1β was measured in MVs and plasma by ELISA (RND systems, DY401) according to the manufacturer’s instructions. Protein concentrations of MVs and plasma were assessed using a BCA protein determination kit (Thermo Fischer #23225). HMGB1 and IL-1β measurements in MVs were normalized to total MV protein to account for sample variation. Previously in our laboratory we have observed that there is a Hook effect when measuring HMGB1 in plasma samples, which results in erroneously low plasma HMGB1 measurements when a low dilution factor is used. Therefore, a dilution factor of 30, optimized from our previous work, was used for all plasma HMGB1 ELISA assessments.

### Co-immunoprecipitation

Co-immunoprecipitation to assess for HMGB1/IL-1β complexes in plasma was performed using the Catch and Release^®^ kit from Millipore according to the manufacturer’s instructions. Briefly, 500μg of sample was incubated in the spin column with anti-HMGB1 antibody and the included antibody capture affinity ligand for 30 minutes. The flow through was collected by centrifugation of the spin column. The column underwent multiple washes followed by elution. The eluate was assessed by Western Blot for HMGB1 and IL-1β.

### Western blot

Western blot was performed as described previously [17]. Eluate from co-IP were run on 4–15% Ready Gel Tris-HCL gel (BioRad), and transferred onto PVDF membranes (BioRad). Membranes were incubated overnight at 4°C with primary antibody. Secondary incubation was performed the following day, and membranes visualized and bands quantified using the LiCor Odyssey imaging system^TM^.

### Flow cytometry analysis of microvesicles

Composition of MVs was done as we have previously reported [17, 41, 42]. Briefly, pelleted MVs were permeabilized with Fix/Perm buffer (Biolegend) for 20 minutes at room temperature. MVs were pelleted at 20,000g for 30 min, incubated with primary antibodies for 20min at 4°C: PE-conjugated HMGB1 antibody (Novus, NBP2-27400PE) and an APC-conjugated IL-1β antibody (eBioscience, #47–7114). After staining, MVs were pelleted and resuspended in PBS. The Stratedigm S1000Ex was used to assess the stained MVs at the UNC Flow Cytometry Core Facility. Size gates to identify MVs (0.1–1.0μm) were set using MegaMix^TM^ (BioCytex) size gating beads (S1A Fig). Single color controls for each primary antibody, compared to unstained media, were used to develop the compensation matrix and distinguish background staining from specific staining using *FlowJo*^TM^ software version 10.0 as described previously [17]. Isotype controls resulted in minimal staining, consistent with recognition of the specific antigens. MV counts from fluorescent events were calculated using AccuCount^TM^ beads (SpheroTech, Inc.) in each sample per the manufacturer’s instructions. Representative histograms of unstained and either PE (anti-HMGB1) or FITC (anti-IL-1β) vesicle populations are depicted in S1B Fig.

### THP-1 cell culture

THP-1 human monocyte cells [43] (obtained from the UNC-Chapel Hill Tissue Culture Facility Repository) were allowed to grow in culture as described previously in standard cell culture conditions in RPMI media containing, 10% fetal bovine serum, 1% penicillin/streptomycin, and 0.05mM 2-mercaptoethanol [30, 44]. Approximately 3x10^5^ cells were plated in 6-well Corning^TM^ cell culture plates. Cells were treated for 24 hours and then harvested for analyses.

### Isolation of mRNA and quantification via RT-PCR

Isolation of mRNA was performed as previously [45]. Briefly, THP-1 macrophages were lysed with TRIZOL buffer. Total RNA was isolated using RNeasy Mini Kit (Qiagen Inc., CA). RNA quantification was performed using a nanodrop 2000^TM^ spectrophotometer. For reverse transcription, 2μg of RNA was used to synthesize cDNA using random primers (Invitrogen) and reverse transcriptase Moloney murine leukemia virus (Invitrogen). The primer sequences used for reverse transcriptase of mRNA targets are included in Table 1. Genes of interest were normalized to the housekeeping gene β actin. Melt curves were assessed for single peaks to identify primers that yielded a single PCR product.

**Table 1:** Primers used for RT-PCR quantification.

Target	Forward (5’ to 3’)	Reverse (5’ to 3’)
IL-6	ACTCACCTCTTCAGAACGAATTG	CCATCTTTGGAAGGTTCAGGTTG
NOX2	ACCGGGTTTATGATATTCCACCT	GATTTCGACAGACTGGCAAGA
iNOS	TTCAGTATCACAACCTCAGCAAG	TGGACCTGCAAGTTAAAATCCC
IFNβ	GCCGCATTGACCATCTATGAGA	GAGATCTTCAGTTTCGGAGGTAAC
β-actin	CTACAATGAGCTGCGTGTGGC	CAGGTCCAGACGCAGGATGGC

## Results

### HMGB1 and IL-1β are concentrated in plasma microvesicles following mouse burn injury model

**Fig 1:**
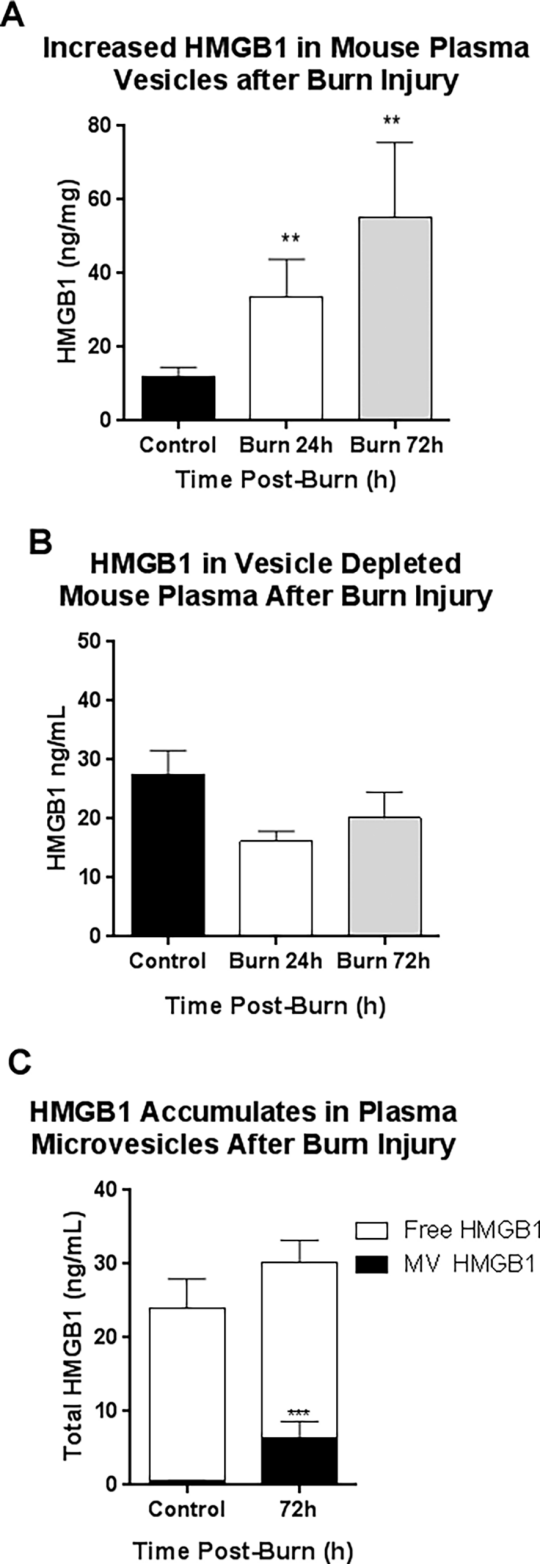
HMGB1 is concentrated in mouse plasma microvesicles (MVs), but not vesicle depleted plasma up to 72 hours after burn injury. Mice underwent a 20% TBSA thermal injury and were sacrificed at either 24 or 72 hours post-burn. Plasma microvesicles (MVs) were isolated from by sequential centrifugation. HMGB1 levels were assessed by ELISA in the MVs and MV-depleted plasma. (A) HMGB1 was increased in plasma MVs up to 4.6-fold after burn injury. This was observed at 24 hours post-burn (33.6±10.2 vs. 12.06±2.43 ng/mg total protein, mean±SEM, Burn vs Control, **p<0.01,) and at 72 hours post-burn (55.35±20.23 vs. 12.06±2.43 ng/mg total protein, Burn vs Control, mean±SEM, **p<0.01) N = 13 control and 5–6 burn mice per group. (B) HMGB1 levels were not increased in the MV-depleted plasma (C) Analysis of plasma fraction, MV versus MV free plasma, showed that plasma HMGB1 increases at 72 hours were due to increases in MV fraction.

HMGB1 and IL-1β are critical mediators of immune responses in many disease settings. Following burn injury HMGB1 and IL-1β are released in plasma [10, 14, 46, 47]. We and others have shown previously that HMGB1 is released in microvesicles (MVs) in response to immune activation [15, 17, 42]. Thus, we hypothesized that both HMGB1 and IL-1β would be released in plasma MVs after large burn injury, and that MVs would serve as a reservoir for these immune molecules. Mice underwent a 20% TBSA thermal injury as described previously [48]. Mice do not become septic and receive fluid resuscitation and pain medication after injury. Plasma was collected during the acute (24h) and sub-acute (72h) time periods. HMGB1 was measured in both plasma MVs, and MV-depleted plasma. Burn injury significantly increased HMGB1 concentration in MVs, but not in MV-depleted plasma. Interestingly, the concentration of HMGB1 in MVs increased with time with a 2.8-fold increase at 24h and a 4.6-fold increase at 72h after burn injury (Fig 1A, *p<0.05). MV-depleted plasma did not show an increase in HMGB1, rather a non-significant trend toward a decrease (Fig 1B). By 72h post-burn, eschar formation over the burn has already occurred, suggesting active vesicular secretion rather than passive release from dying cells. An analysis of the distribution of HMGB1 (i.e. either in plasma MVs or in MV-free plasma) showed that at 72h, the increase in plasma HMGB1 was completely localized to MVs (Fig 1C). Thus, plasma MVs serve as a reservoir for HMGB1 after burn injury.

**Fig 2:**
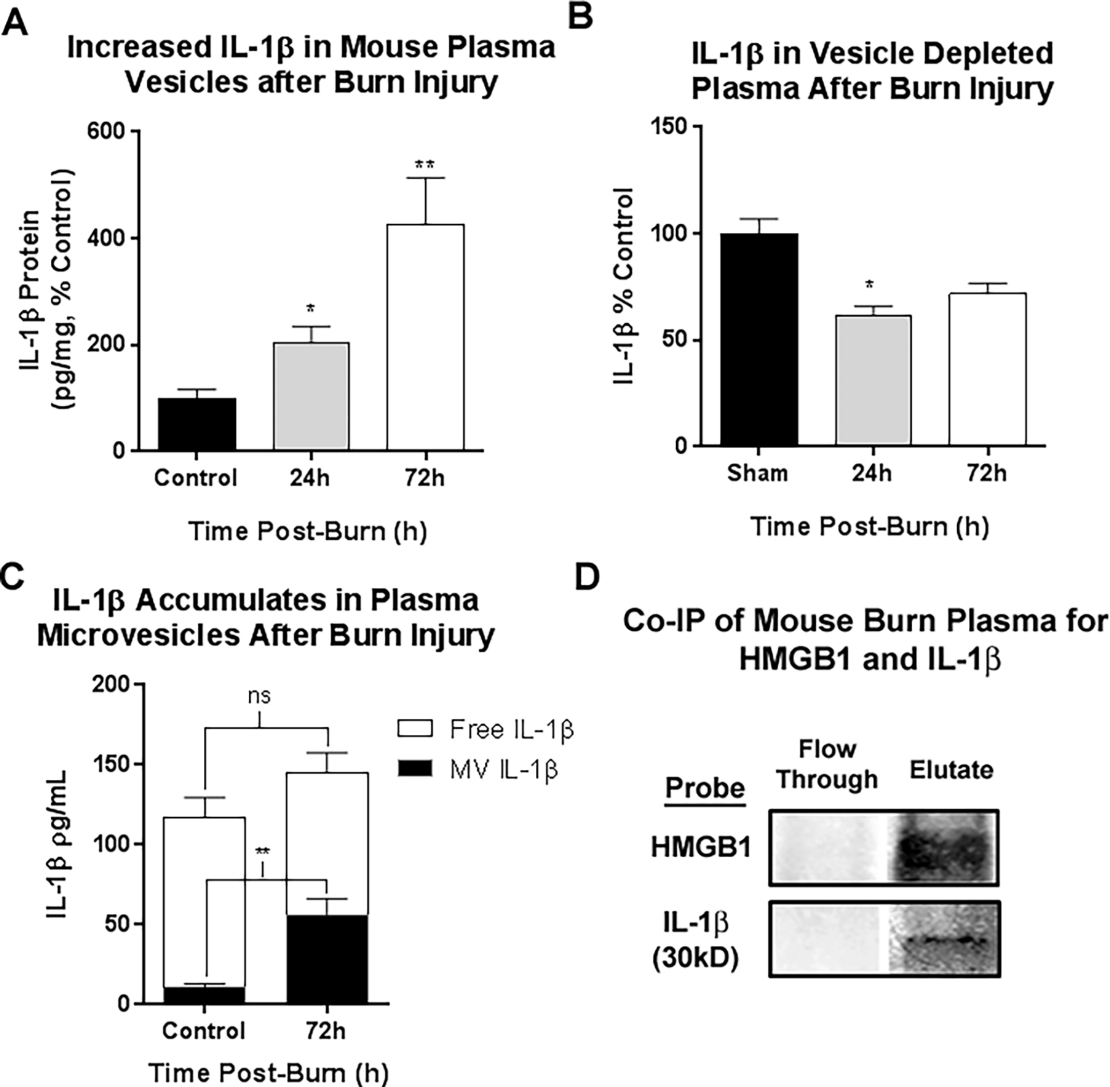
IL-1β is concentrated in mouse plasma microvesicles (MVs), but not vesicle depleted plasma up to 72 hours after burn injury. Mice underwent a 20% TBSA thermal injury and were sacrificed at either 24 or 72 hours post-burn. Plasma microvesicles (MVs) were isolated from by sequential centrifugation. IL-1β levels were assessed by ELISA in the MVs and MV-depleted plasma. (A) IL-1β was increased in plasma MVs up to 426% of control levels. At 24 hours post-burn IL-1β was 2-fold control levels; 203.1±32.1 vs. 100±10.7% control, mean±SEM, Burn vs Control, group *p<0.05, N = 3 control, 4 burn. At 72 hours post-burn IL-1β levels in MVs were 400% control values; 426.1±88.09 vs. 100 ± 10.7% control, Burn vs Control, mean±SEM, **p<0.01, N = 5 per group. Data from two separate experiments were combined and presented as percent control. (B) In MV-depleted plasma, IL-1β levels were not increased. At 24 hours, MV-free IL-1β was significantly reduced by 21%; 100±8.43% vs 79.12±4.02%, mean±SEM, Control vs Burn, *p*<0.05, N = 4–5. At 72 hours post burn a nonsignificant reduction in IL-1β was observed; 100±10.10% vs 80.43±12.36%, mean±SEM, Control vs Burn, *p* = 0.24, N = 5–7. (C) Analysis of plasma fraction, MV versus MV free plasma, showed that plasma IL-1β increases at 72 hours were due to increases in MV fraction. (D) Co-immunoprecipitation (Co-IP) of mouse plasma for HMGB1 was performed. Column flow through and eluate were assessed by western blot for HMGB1 and IL-1β. Both HMGB1 and IL-1β were found in the eluate indicating HMGB1 and IL-1β complex formation in mouse plasma. Both the 31kD pro-form of IL-1β and HMGB1 were detected in the eluate, but not the flow through demonstrating the presence of HMGB1/pro-IL-1β complexes in human plasma.

IL-1β is often considered a companion cytokine for HMGB1. Both HMGB1 and IL-1β can be secreted from cells in vesicles, and utilize common vesicular secretory pathways [25, 49]. We found that IL-1β was increased in plasma MVs after burn injury. At 24h post-burn, IL-1β was increased in MVs by 203% above control levels (**p<0.01, Fig 2A). At 72 hours after injury, IL-1β in MVs was increased even further, up to 426% of control levels. IL-1β in vesicle-depleted plasma, however, was significantly reduced to 73% of control at 24h (*p<0.05, Fig 2B). Analysis of IL-1β localization at 72 hours post-burn showed that the increase in IL-1β was completely due its increase in MVs and not MV-free IL-1β (Fig 2C). Thus, similar to HMGB1, IL-1β is persistently concentrated MVs after burn injury. This indicates that microvesicles serve as a reservoir for both IL-1β and HMGB1 after burn injury. We found recently in mice that HMGB1 and IL-1β form heterocomplexes after immune activation [50]. Others have shown previously that HMGB1 and IL-1β form potent heterocomplexes *in vitro* [12, 13]. The higher molecular weight complexes we detected were stable, detected by western blot (~65kD), co-immunoprecipitation, and disappeared following siRNA directed against HMGB1 [50]. Thus, we hypothesized that HMGB1 and IL-1β would form heterocomplexes in plasma after burn injury. Western blot analysis of plasma microvesicles found free HMGB1, free IL-1β and higher molecular weight HMGB1/IL-1+ bands (S2A–S2C Fig) consistent with our previously published findings. There was a measured increase in the ~65kD HMGB1/IL-1β+ expression in plasma MVs after burn injury (S2D Fig, *p<0.05, 53-fold increase, N = 6 per group), while there was no change in free HMGB1 (S2D Fig, *p =* 0.68). In order to confirm the presence of HMGB1/IL-1β complexes we performed co-immunoprecipitation on mouse plasma. After immunoprecipitation for HMGB1, the 31kD pro-IL-1β was found in the eluate consistent with stable HMGB1/pro-IL-1β complexes in the plasma (Fig 2D and S3 Fig). Thus, both HMGB1 and IL-1β are concentrated in plasma MVs after burn, and HMGB1 and pro-IL-1β form stable heterocomplexes in plasma MVs.

### Increased co-localization of HMGB1 and IL-1β in plasma microvesicles following burn injury in mice

**Fig 3:**
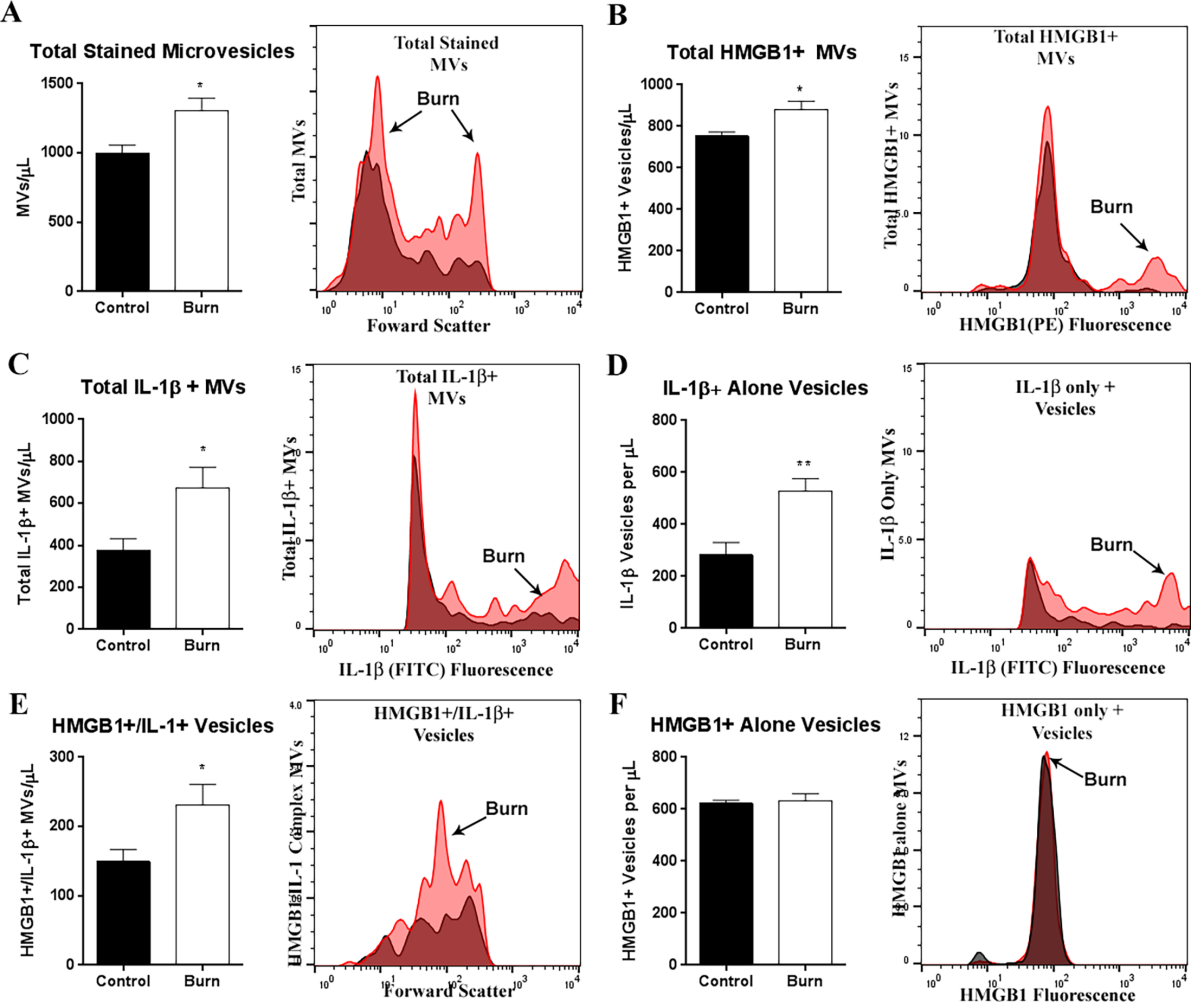
HMGB1 and IL-1β Co-localize in plasma microvesicles (MVs) following burn injury. Mice underwent a 20% TBSA thermal injury and were sacrificed at 72 hours post-burn. Plasma microvesicles (MVs) were isolated from by sequential centrifugation. Vesicles were permeabilized using Fix/Perm buffer and labeled with anti-HMGB1 and anti-IL-1β antibodies. MVs between 0.1–1.0μm were identified using MegaMix size gating beads. Positive staining was differentiated from background by comparing to single color controls for each antibody. (A) The total number of stained MVs was increased by 34% at 72 hours after burn injury; **p<0.01, N = 8 control, 6 burn. (B) The total number of HMGB1+ MVs was increased by 17%; 753.5 ± 19.6 vs 879.1 ± 41.7; Control vs Burn N = 7 Control, 6 Burn (C) The total number of IL-1+MVs was increased by 78%; 377.7 ± 54.9 vs 672.5 ± 99.1 Control vs Burn N = 8 Control, 6 Burn. (D) MVs positive for IL-1β alone were increased after burn injury by 61%; 281.9 ± 51.22 vs 536.6 ± 45.76, Control vs Burn, *p<0.05, N = 7 control, 5 burn. (E) MVs positive for both HMGB1 and IL-1β were increased by 64%; 145.4 ± 15.30 vs 237.8 ± 32.74, Control vs Burn, *p<0.05, N = 7 control, 6 burn. (F) MVs positive for HMGB1 alone were not changed after burn injury, 612.7 ± 13.27 vs 641.3 ± 22.73, Control vs Burn, *p* = 0.27, N = 8 control, N = 6 burn. Representative relative frequency histograms depicting different MV populations for each panel are included: red-burn, white-control. **p<0.01, *p<*0.05, *t-*test vs control.

**Fig 4:**
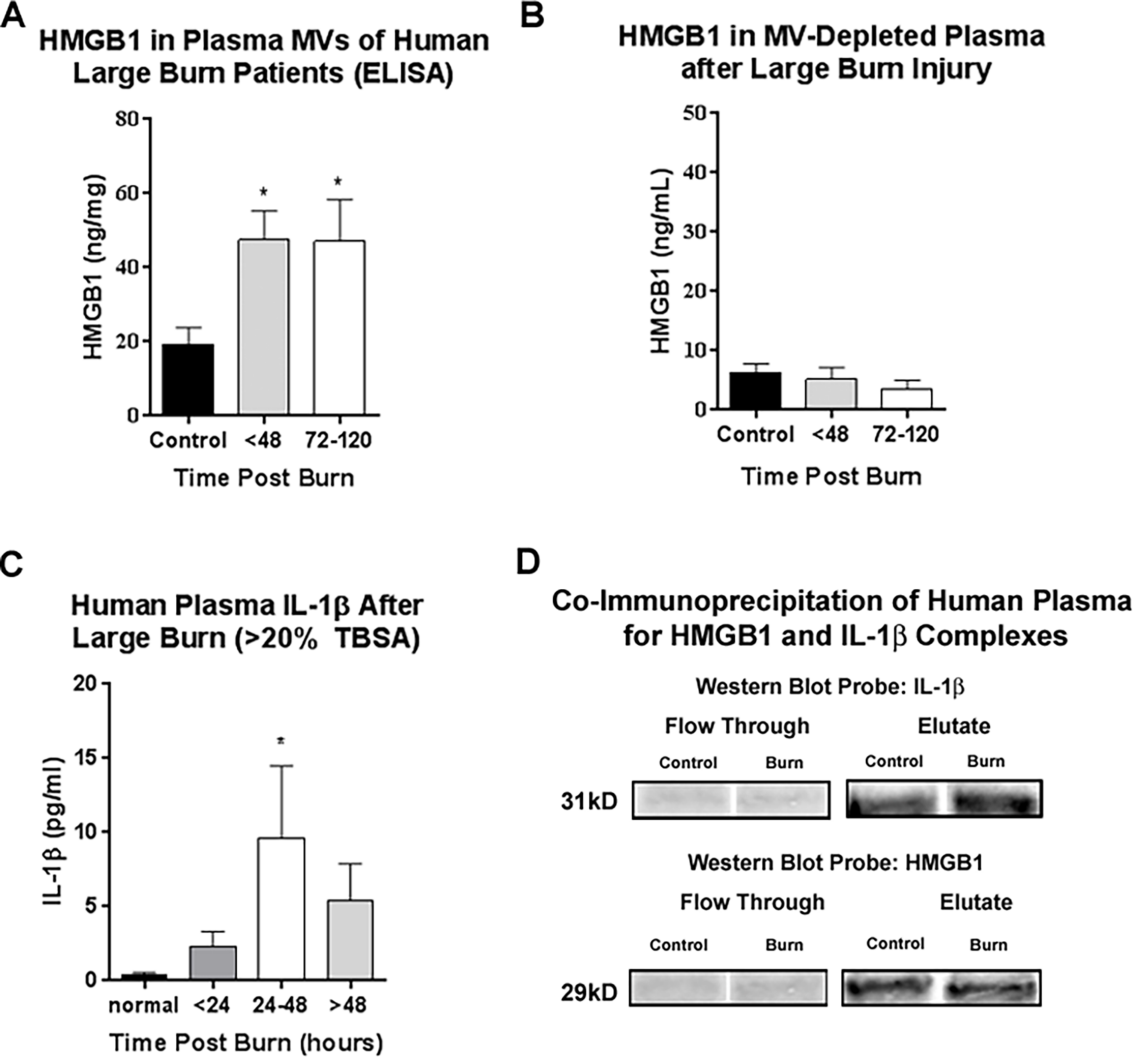
Human large burn injuries cause increased HMGB1 release in microvesicles (MVs), IL-β, and HMGB1/IL-1 complexes in plasma. In two different patient groups of adult patients admitted to the North Carolina Jaycee Burn Center at the University of North Carolina at Chapel Hill hospital were enrolled into the study and serial blood draws were taken. IL-1β and HMGB1 were measured in plasma and plasma MVs respectively. (A-B) Plasma was collected from 15 human burn patients with a mean age of 36.5 (range 12–77); 80% male, mean %TBSA 14.5% (range 1.5–42%). Plasma collections were obtained during the first 48 hours of admission and between 72–120 hours after admission. See Table 2 for details. (A) Microvesicles (MVs) were isolated by centrifugation. HMGB1 was measured in MVs by ELISA. HMGB1 was increased in plasma MVs 2.5-fold after burn injury. This was observed both within 48 hours (47.6±7.73 vs. 19.32±4.5 ng/mg total protein, Burn vs Control) and at 72–120 hours after admission (47.09±19.5 vs. 19.32±4.5 ng/mg total protein, Burn vs Control, mean±SEM, *p<0.05) N = 3–6 per group. (B) HMGB1 was measured in MV-depleted plasma by ELISA. HMGB1 in MV-depleted plasma was unchanged after burn injury. (C-D) Plasma was collected from 22 patients, mean age 42 (range 18–78); 82% male, mean %TBSA 26% (range 5–45%), see Table 3. The first blood sample was obtained at 0–24 hours following initial burn injury, the second was obtained at 24–48 hours following injury, and the third was obtained at 48–72 hours following injury. (C) Large burn (>20% TBSA, n = 8) was associated with a significant (*p < 0.05) increase in IL-1β plasma concentration at 24–48 hours compared to control, normal individuals. (D) Co-immunoprecipitation was performed in human plasma to confirm the presence of HMGB1/IL-1β complexes. Western blot was performed and the flow through and eluate probed for IL-1β and HMGB1. Both the 31kD pro-form of IL-1β and HMGB1 were detected in the eluate, but not the flow through demonstrating the presence of HMGB1/pro-IL-1β complexes in human plasma.

As mentioned above, HMGB1 and IL-1β were both increased and concentrated in MVs after burn injury and formed stable heterocomplexes. Both HMGB1 and IL-1β can share a common unconventional secretion mechanism [25]. This, coupled with our recent observation that HMGB1 and IL-1β form heterocomplexes and colocalize in cytoplasmic vesicular structures following immune activation[50] led us to hypothesize that HMGB1 and IL-1β would be found in plasma MVs together after burn injury. We used flow cytometry to measure HMGB1 and IL-1β in plasma MVs as we and others have described previously [17, 42]. These MVs range in size from 0.1–1.0μm in diameter. Burn injury increased the total number of plasma MVs at 72h post-burn by 34% (*p*<0.01, Fig 3A). A clear increase in two distinct populations of MVs was observed. No difference in total unstained particles was measured (*p =* 0.36, not shown). Consistent with our ELISA measurements, the numbers of total HMGB1 and total IL-1β positive MVs were both increased (16% and 78% respectively, Fig 3B and 3C, *p<0.05). After burn injury, a second population of HMGB1+ vesicles was seen that was not in controls (Fig 3B). This represented HMGB1+ MVs that were also positive for IL-1β. Both the IL-1β + alone and IL-1β/HMGB1 + co-labeled MVs were increased by 61% and 63% respectively (*p*<0.05, Fig 3D and 3E). Interestingly, the number of HMGB1 + alone MVs was unchanged (Fig 3F), though the HMGB1+/IL-1+ population was increased by 63% (Fig 4E). Thus, there is increased secretion of HMGB1 IL-1β in MVs after burn injury. IL-1β, however, can be secreted in vesicles with or without HMGB1. Thus, these observations indicate that both HMGB1 and IL-1β co-localize in MVs together after burn injury, and that persistent secretion of HMGB1 in vesicles occurs with IL-1β.

### HMGB1 and IL-1β are increased in plasma from human burn injury patients

Since we observed that burn injury increases HMGB1/IL-1β complexes in MVs *in vivo*, we then assessed plasma from human burn patients. We assessed HMGB1 and IL-1β in two distinct but similar patient groups from the UNC Jaycee Burn Center (Tables 2 and 3). Similar to our observations in mice, HMGB1 was increased in plasma MVs of human burn patients. HMGB1 was increased in MVs by approximately 2.5-fold (*p<0.05) in burn injury patients both during acute (<48h) and sub-acute periods after burn injury (>72-120h) (Fig 4A). During the first 48 hours, HMGB1 in MVs correlated positively with total %TBSA (R = 0.67, *p =* 0.03, not shown). However, in plasma in which MVs were depleted by centrifugation, HMGB1 was not increased (Fig 4B). This identifies that post-burn HMGB1 is increased primarily in MVs, making MVs a reservoir for HMGB1 after burn injury. IL-1β has previously been found to be elevated for weeks after severe burn injury in humans [9]. We replicated this finding in our second patient population. We found that IL-1β is increased approximately 9-fold in plasma of large burn injury patients during the 24-48h time period post-burn, with a trend to significance prior to 24 hours (Fig 4C, *p<0.05). Small burns (<20% TBSA, n = 4) showed a much lower level of plasma IL-1β, that was significantly increased at 48 hours (0.4 vs 1.6pg/mL, Control vs Burn, *p<0.05, not shown). Since HMGB1 and IL-1β have been shown to form complexes *in vitro* [12] and we found HMGB1/IL-1β complexes in mouse burn plasma, we assessed the human plasma. Co-immunoprecipitation of human plasma for HMGB1 and IL-1β found that both HMGB1 and IL-1β were found in the eluate by western blot (Fig 4D and S4 Fig) consistent with HMGB1/IL-1β complexes in human plasma. As with the mouse plasma, the detected IL-1β was the 31kD pro-form. These data indicate that burn injury causes the release of HMGB1 in plasma MVs. HMGB1 forms complexes with pro-IL-1β complexes in human plasma, similar to observations in mice.

**Table 2:** Detailed demographic and outcomes data for human burn patients for HMGB1 ELISA assessments. Control Patients: n = 5, age- and sex-matched, healthy volunteers. *TBSA*, total body surface area; *DC*, discharged.

Patient	Age	Gender	TBSA (%)	Degree	Length of Stay (days)	Outcome
A	32	M	1.5	2nd	1	DC
B	26	M	2	2nd	3	DC
C	12	M	2	2nd	6	DC
D	56	M	2	2nd	11	DC
E	44	F	3	2nd	4	DC
F	22	M	4	3rd	4	DC
G	34	F	7	2nd	10	DC
H	12	M	15	3rd	19	DC
I	45	M	15	3rd	43	DC
J	34	M	20.5	3rd	21	DC
K	31	M	21	3rd	64	DC
M	76	F	21	3rd	68	DC
N	77	M	26.5	3rd	120	Death
O	18	M	35	2nd	92	DC
P	29	M	42	3rd	76	DC

**Table 3:** Detailed demographic and outcomes data for human burn patients for IL-1β. Control Patients: n = 10, age- and sex-matched, healthy volunteers. *TBSA*, total body surface area; *DC*, discharged.

Patient	Age	Gender	TBSA (%)	Degree	Length of Stay (days)	Outcome
A	33	M	7	2nd	7	DC
B	34	M	8	3rd	19	DC
C	45	M	12.5	2nd	44	DC
D	27	M	19	2nd	33	DC
E	77	F	30	3rd	48	DC
F	77	M	37	3rd	114	Death
G	52	F	40	2nd	143	DC
H	50	M	41	2nd	11	DC
I	52	M	41.5	3rd	56	DC
J	18	M	42	3rd	57	DC
K	41	M	47	3rd	68	DC
L	20	M	53	3rd	38	DC
M	23	M	14	3rd	56	DC
N	56	M	16	2nd	23	DC
O	78	F	18	2nd	46	Death
P	45	M	5	3rd	4	DC
Q	18	M	45	2nd	22	DC
R	33	F	18	3rd	8	DC
S	56	M	31	3rd	78	Death
T	54	M	32	2nd	45	DC
U	19	M	16	3rd	13	DC
V	24	M	8	2nd	12	DC

### HMGB1/IL-1β complexes enhance immune responses in THP-1 monocytes, similar to findings in burn injured patients

**Fig 5:**
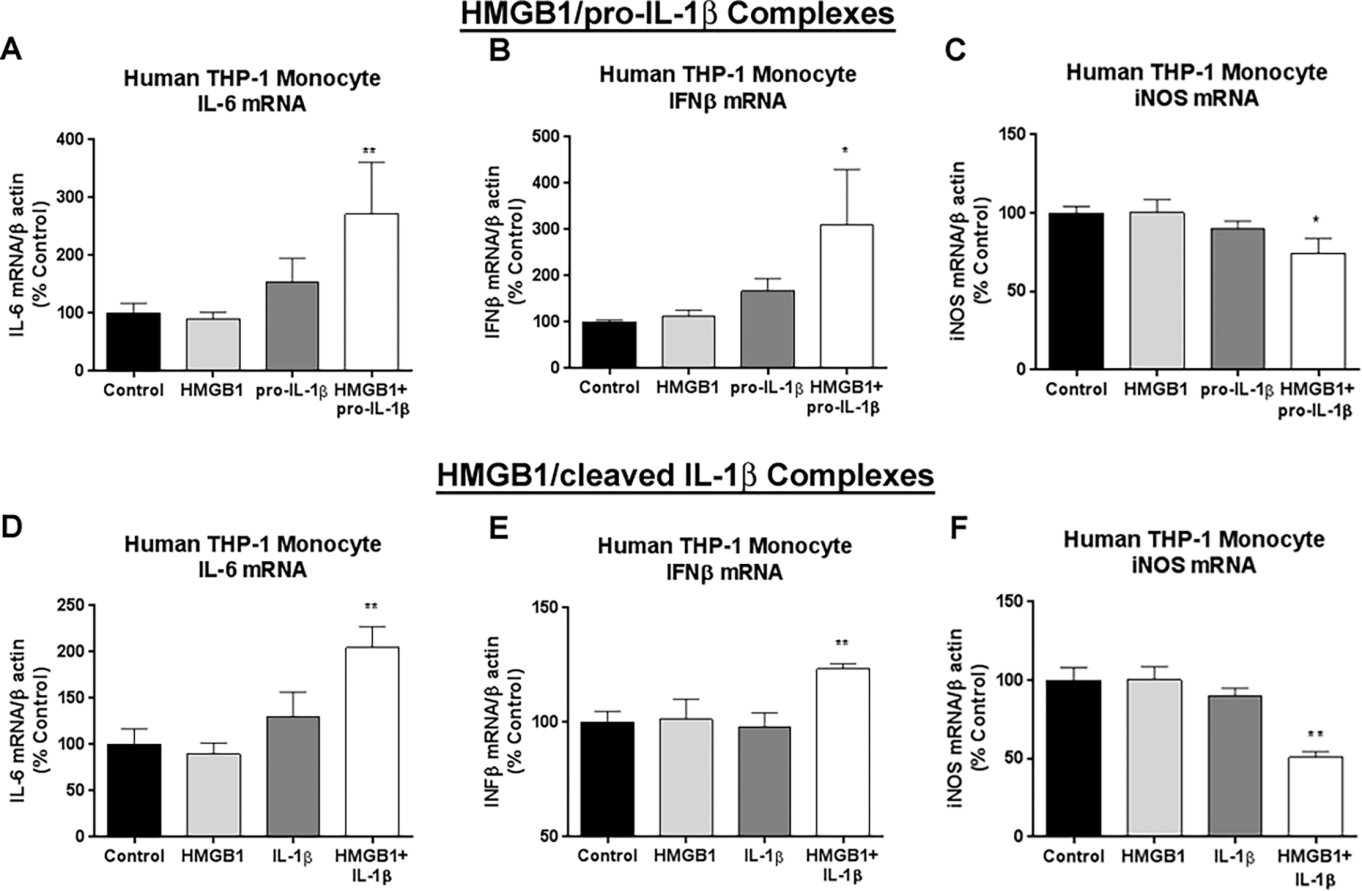
Enhanced induction of proinflammatory gene expression by HMGB1/IL-1β complex. Human THP-1 monocytes were incubated with either recombinant (r) human HMGB1 (4nM, 100ng/mL), recombinant pro-IL-1β (rpro-IL-1β, 4nM) or rHMGB1/r pro-IL-1β complexes. Cell lysates were harvested 24 hours later and analyzed by RT-PCR for expression of immune gene of interest. (A) Neither HMGB1 nor pro-IL-1β alone significantly altered IL-6 mRNA. However, rHMGB1/r pro-IL-1β complexes caused a 2.7-fold increase in IL-6 gene expression (B) Neither HMGB1 nor pro-IL-1β alone significantly altered IFNβ mRNA. rHMGB1/r pro-IL-1β complexes caused a 3.1-fold increase in IFNβ gene expression (C) rHMGB1/rpro-IL-1β caused a 36% reduction in iNOS gene expression, while neither HMGB1 nor pro-IL-1β alone caused changes (D) r-pro-IL-1β caused a 2.5-fold increase in NOX-2 alone. rHMGB1/rIL-1β complexes showed no further increase in NOX-2 gene expression. ***p<0.001, **p<0.01, *p<0.05 vs control. N = 2–6 culture wells per group.

**Fig 6:**
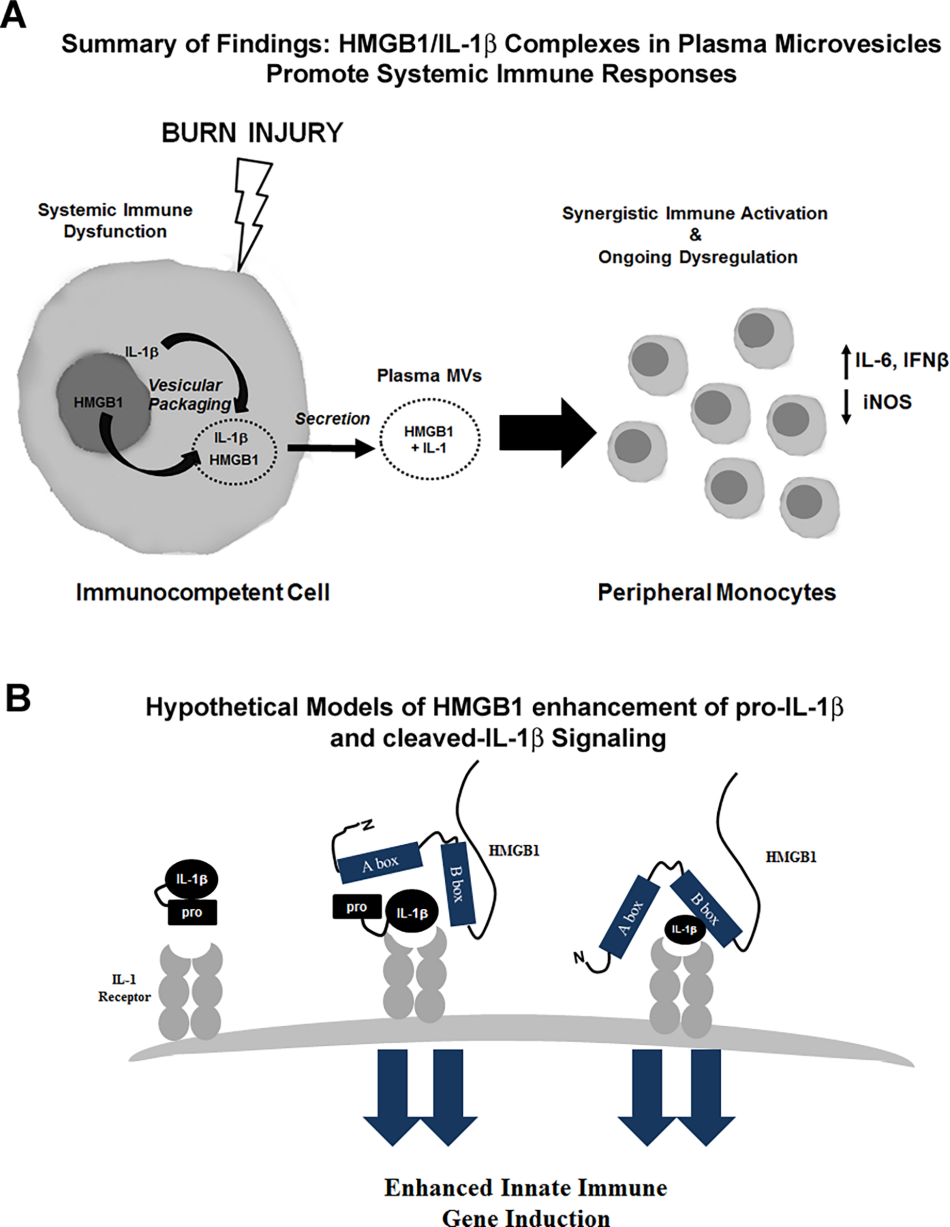
Proposed model of enhanced activation by HMGB1/IL-1β complexes. (A) Schematic illustrating secretion of HMGB1 and IL-1 after burn injury. HMGB1 translocates from the nucleus to the cytoplasm where it is packaged in vesicles. HMGB1 and IL-1β were increased in plasma MVs after burn injury. HMGB1/IL-1β complexes caused enhanced increases in IL-6 and IFNβ gene induction in human THP-1 monocytes. (B) Hypothetical models of enhanced immune activation by HMGB1/pro-IL-1β and HMGB1/cleaved-IL-1β complexes. Pro-IL-1β is considered inactive at the IL-1 receptor. When coupled with HMGB1, pro-IL-1β caused enhanced immune activation in a manner similar to HMGB1/cleaved IL-1β complexes.

Since we identified that burn injury causes an increase in HMGB1 and IL-1β complexes, we next investigated to biological activity of these complexes. Large burn injuries are associated with prolonged immune dysfunction. This includes persistent immune activation (seen by persistently elevated IL-6) [10, 47] as well persistent immune impairment (seen by reduced iNOS activation) [34] and reduced macrophage clearance of pathogenic bacteria [8, 51]. IL-6 is a key burn-associated cytokine that correlates best with mortality [10]. We investigated whether HMGB1 and IL-1β complexes altered monocyte activation. In order to ensure the concentration of complexes used the same across all experimental groups, we used exogenous HMGB1/IL-1β complexes formed from recombinant proteins to model the clinical condition. Using human THP-1 monocytes, we assessed the effect of rHMGB1/rIL-1β complexes on innate immune gene activation. Since the pro-form of IL-1β was found in complexes with HMGB1 in humans and mice by co-immunoprecipitation, we prepared complexes with recombinant pro-IL-1β (31kD). Using western blot, we confirmed that the recombinant pro-IL-1β was not contaminated with the cleaved form (not shown). Complexes were formed by incubation at 4°C for 24h as described previously [12]. THP-1 monocytes were then incubated with either rHMGB1 alone (4nM), rpro-IL-1β alone or rHMGB1/rpro-IL-1β complexes at the same concentrations for a 1:1 molar ratio of the two molecules. These concentrations of rHMGB1 and r-pro-IL-1β alone did not produce any measureable gene transcription changes in IL-6, iNOS, or IFNβ (Fig 5A–5D). The addition of the rHMGB1/rIL-1β (31kD) complexes, however, caused enhanced increases in IL-6 (271% vs control, *p<0.05 Fig 6A) and IFNβ (310% vs control, *p<0.05). Interestingly, HMGB1/IL-β complexes resulted in reduced iNOS gene transcription by 50% (Fig 5C), consistent with our previous observation of reduced plasma nitric oxide after burn injury [34]. Thus, rHMGB1/r-pro-IL-1β complexes produce innate immune gene changes in human THP-1 monocytes similar to those seen after burn injury. Pro-IL-1β, which is considered inactive at IL-1β receptors, becomes active and potent when complexed with HMGB1. HMGB1 has been previously found to form complexes with cleaved IL-1β *in vitro* [12, 13], thus we also assessed HMGB1/IL-1β as a comparison. The pattern of innate immune gene activation by HMGB1/cleaved-IL-1β was very similar to that of HMGB1/pro-IL-1β complexes. Enhancement of IL-6, IFNβ and suppression of iNOS were all seen (Fig 5D–5F). These complexes have immune-stimulating activity, with a similar activation pattern to that observed in burn injuries.

## Discussion

The regulation of immune responses to burn injury or sepsis is complex and includes multiple immune factors. Large burn injuries are associated with an initial sepsis-like immune activation [7–10]. This is followed by a persistent immune dysregulation that causes increased susceptibility of bacterial and fungal infections. We found that HMGB1 and IL-1β are released in microvesicles after acute burn injury and form immune-modulating complexes. DAMP release immediately after a large burn injury is profound and likely contributes to the initial systemic immune responses. HMGB1 inhibition with glycyrrhizin was found to reduce IL-1β and TNFα responses at 48h post-burn [52]. We observed ongoing release of HMGB1 in MVs up to 3–5 days after injury, a time in humans when wound debriding and skin grafting have occurred, and mouse burn injuries have been covered with granulation tissue. Thus, the source of the initial DAMP release, the damaged tissue, has been removed. This suggests an ongoing active release of HMGB1 either from newly grafted/formed tissue or from other peripheral immune cells. Our findings suggest this ongoing release regulates subsequent immune function.

We found here that HMGB1 couples with pro-IL-1β in plasma and modulates immune responses in human THP-1 monocytes. Previous studies have found that HMGB1 can form complexes with cleaved IL-1β, *in vitro*, enhancing its activity [13]. We showed recently in mouse tissue and in human brain tissue that HMGB1 can form complexes with the pro- and cleaved forms of IL-1β [50]. We now report that when HMGB1 was coupled with pro-IL-1β, pro-IL-1β becomes active. This is a novel signaling function for pro-IL-1β. HMGB1/pro-IL-1β complexes showed similar activity to HMGB1/cleaved IL-1β complexes *in vitro*. We used exogenously formed complexes in these experiments to ensure consistent concentrations of each molecule. However, future studies should investigate the efficacy of endogenous complexes. This will require development of methods to isolate endogenous complexes with high purity. Complexes lead to enhanced IL-6 and IFNβ responses, both of which are induced in burn injury patients. IL-6 is one of the key cytokines that is persistently increased following large burn injury that correlates with mortality [10, 47]. Burn sepsis is associated with a combination of both immune hyper-activity, as well as suppression. In addition to its antiviral activities, IFNβ has been identified as a key mediator of immunosuppression that increases susceptibility to bacterial infection. IFNβ inhibits IL-1β production [53, 54] and inhibits iNOS production [54]. Our observation of reduced iNOS mRNA following HMGB1/IL-1β complexes is consistent with our previous finding of reduced plasma NO following burn injury [34] and may suggest impaired phagocytic activity. Thus, the ongoing release these complexes may to be involved in post-burn immune dysfunction through effects on monocytes. It is currently unclear how binding to HMGB1 alters the activity of pro-IL-1β. Pro-IL-1β is typically thought to be inactive at the IL-1β receptor [55]. We find that pro-IL-1β becomes active when bound to HMGB1. The precursor domain of pro-IL-1β is thought to prevent the full barrel formation associated with the constitutively active cleaved IL-1β [56]. Binding to HMGB1 could potentially expose this active site (Fig 6B) or facilitate its interaction with membrane bound caspase-1, though this mechanism needs further elucidation. Since the concentrations of HMGB1 used did not show effects alone, we suspect that TLR4 or RAGE activation by HMGB1 is not responsible for our findings. Further, we and others have shown previously that the enhancement of signaling by HMGB1/cleaved IL-1β heterocomplexes *in vitro* is via the IL-1β receptor [11–13, 50]. However, the involvement of TLR4 or RAGE cannot be entirely ruled out. Nonetheless, we find a novel role of immune induction by pro-IL-1β. HMGB1/pro-IL-1β complexes are increased in the plasma after burn injury and modulate immune responses of monocytes.

Our findings identify that microvesicles play a key role in burn immune pathology. Microvesicles have been identified as key players in multiple disease pathologies such as cancer [24, 57], inflammatory arthropathies [58, 59], lupus [21, 60], renal injury [61, 62] and sepsis [63, 64]. MVs can have exert many effects on target cells, via delivery of DAMPS, cytokines or miRNA [21]. Though miRNA in MVs was not investigated in this study, their role in modulating target cells is likely important and should be investigated. Microvesicles from leukocytes, granulocytes, monocytes and endothelial cells have been shown to be increased in human burn patients with and without severe sepsis [64]. Microvesicle numbers correlate with adverse outcomes in sepsis and burn patients, but the detrimental MV-associated molecular mediators are not fully known. We found both HMGB1 and IL-1β were enriched in plasma microvesicles, but not in vesicle-free plasma. This suggests that MVs are a reservoir of HMGB1 and IL-1β and act as key mediators of immune responses associated with these molecules. In our experiments, we lysed vesicles prior to western blot analysis and permeabilized prior to flow cytometry, thus these mediators could be located either on the MV surface or within the MVs. We suspect that HMGB1 and IL-1β bind in vesicles as they are packaged for secretion; however this needs to be elucidated further and we predict that MV/HMGB1 levels will correlate with poor patient outcome, increased immune dysfunction and infection susceptibility in a large cohort of patients.

Our preparation of MVs isolates vesicles between 0.1–1μm in diameter. The origin of these vesicles may include either budding of the plasma membrane, release of secretory autophagomes, or smaller apoptotic bodies [65]. Both HMGB1 and IL-1β utilize the same unconventional vesicular secretion mechanisms, as they lack a leader sequence to target them to the Golgi apparatus [25, 26]. IL-1β, however, can also be released in MVs shed directly from the cell membrane [30, 31]. This might explain why we also observed an increase in a population of IL-1+ MVs that did not contain HMGB1 in addition to the HMGB1+/IL-1+ population. We were intrigued to find HMGB1 bound to pro-IL-1β (31kD) rather than cleaved IL-1β (17kD) in humans and *in vivo*, as the cleaved form is often considered to be the secreted form of IL-1β. Pro-IL-1β, however, has been shown to be secreted in MVs previously *in vitro* [30, 31]. Antibodies used for flow cytometry and ELISAs likely detect both pro and cleaved IL-1β, since the epitope is in the cleaved portion of the protein. This may make it difficult to determine exactly which form of IL-1β is present for diagnostic or therapeutic purposes. Furthermore, our previous findings suggest that HMGB1/IL-1β heterocomplexes are not readily detected by traditional IL-1β ELISAs [29]. This should be considered when performing human studies that measure IL-1β levels. We used co-immunoprecipitation to detect complex formation in human and mouse plasma. Other imaging techniques such as BRET give more direct proof of complex formation in situ. However, for assessment of human plasma samples, co-IP remains the best way to detect co-localization. Our findings strongly suggest the presence of HMGB1/pro-IL-1β complexes in human burn plasma. Thus, the contribution of MVs in immune pathologies should be examined further. The removal of detrimental MVs could serve as a therapeutic approach in the years ahead.

Other laboratories also find that microvesicles are important in sepsis pathologies. Several years ago, endotoxin was found to transiently increase microvesicle numbers in plasma and to be associated with mortality [66, 67]. Since that time it has been found that microvesicles originate from multiple cell types including platelets, leukocytes, granulocytes, erythrocytes, monocytes and endothelial cells [65]. These MVs regulate several functions. For instance, tissue-factor containing MVs are released in patients with sepsis or certain cancers can regulate coagulation state of an individual [68]. Polymorphonuclear leukocytes exposed to staph aureus release MVs that impair bacterial growth [69, 70]. In other settings, MVs can aid in processes such as tissue repair [71]. Mesenchymal stem cell-derived vesicles can protect against mortality in models of acute kidney injury [72]. Thus, depending on the setting and the cargo, MVs can either be detrimental or beneficial. Future studies should continue to assess MV contents in specific disease settings, and how these modulate function.

Our findings indicate that the interaction between cytokines and DAMPs is key in the systemic immune response following burn injury. HMGB1 and pro-IL-1β form complexes in microvesicles to regulate immune responses. Future studies should investigate the potential interactions of other DAMPs and cytokines, as well as other roles of microvesicles.

## Supporting information

S1 Fig Flow cytometry assessment of microvesicles. Microvesicles (MVs) were isolated by size using centrifugation (21,000g for 1 hour) and analyzed by flow cytometry. (A) Depiction of size gating for microvesicles (0.1 to 1.0μm) using MegaMix^TM^ gating beads. (B) Specific staining for cell-type markers was determined by comparison with unstained controls. Depictions of unstained (gray) and either PE (anti-HMGB1) or FITC (anti-IL-1β) vesicle populations for each primary antibody are shown (arrows).
(TIF)

S2 Fig Full length western blot of mouse plasma microvesicles for HMGB1 and IL-1β. Microvesicles (MVs) were isolated by size using centrifugation (21,000g for 1 hour) and analyzed by Western Blot. (A) Probing for HMGB1 revealed three clear bands at 29kD (free HMGB1), approximately 55kD, and 65kD. (B) Probing for IL-1β revealed three clear bands at 31kD (pro-IL-1β), approximately 55kD, and 65kD. (C) Overlay of the staining for HMGB1 and IL-1β showed a separation of the 29 (free HMGB1) and 31kD (pro-IL-1β) bands but overlay of the 55kD and 65kD HMGB1 and IL-1β positive bands.
(TIF)

S3 Fig Entire western blots for co-immunoprecipitation of HMGB1 and IL-1β in mouse plasma. Co-immunoprecipitation was performed for HMGB1 and IL-1β in mouse control and burn plasma. Entire eluate and flow through for these samples are presented. (A) Entire blots of eluates probed for IL-1β and for HMGB1. Bands positive for IgG and pro-IL-1β as well as the Catch and Release® affinity ligand were observed. The blot probed for HMGB1 showed a broad band consistent with free HMGB1 near 29kD. (B) Entire western blots of flow through. Minimal IL-1β and HMGB1 were found in the flow through.
(TIF)

S4 Fig Entire western blots for co-immunoprecipitation of HMGB1 and IL-1β in human plasma. Co-immunoprecipitation was performed for HMGB1 and IL-1β in human control and burn plasma. Entire eluate and flow through for these samples are presented. (A) Entire blots of eluates probed for IL-1β and for HMGB1. Bands positive for IgG and pro-IL-1β as well as the Catch and Release® affinity ligand were observed. The blot probed for HMGB1 showed a broad band consistent with free HMGB1 near 29kD. (B) Entire western blots of flow through. Minimal pro-IL-1β and HMGB1 were found in the flow through.
(TIF)
