# Supplementary figures and images for: HMGB1/IL-1β complexes in plasma microvesicles modulate immune responses to burn injury
Source: PLoS One. 2018 Mar 30;13(3):e0195335. doi: 10.1371/journal.pone.0195335 (PMC5877880; doi:10.1371/journal.pone.0195335)

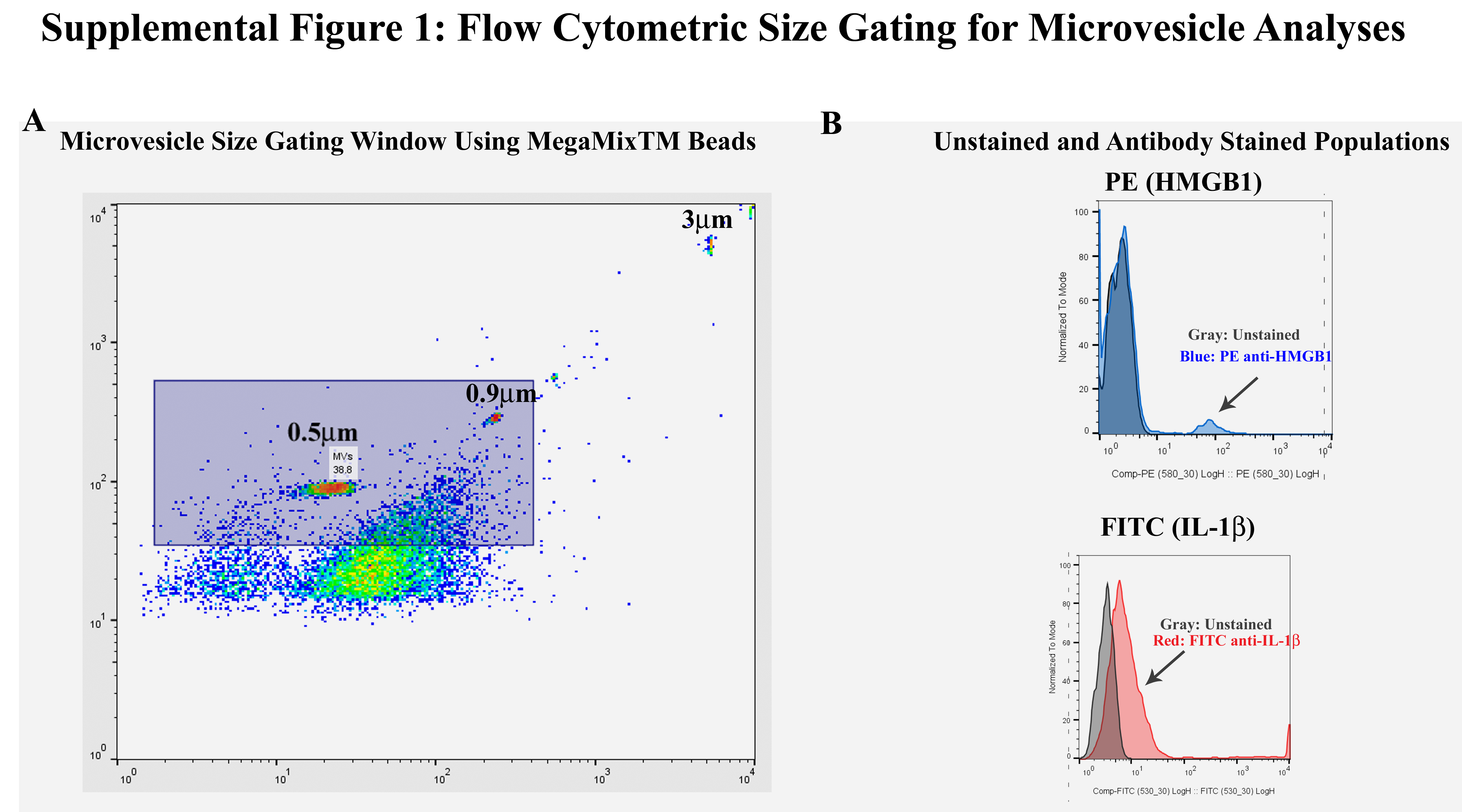

Supplement: S1 Fig — Microvesicles (MVs) were isolated by size using centrifugation (21,000g for 1 hour) and analyzed by flow cytometry. (A) Depiction of size gating for microvesicles (0.1 to 1.0μm) using MegaMixTM gating beads. (B) Specific staining for cell-type markers was determined by comparison with unstained controls. Depictions of unstained (gray) and either PE (anti-HMGB1) or FITC (anti-IL-1β) vesicle populations for each primary antibody are shown (arrows). (TIF) [file pone.0195335.s001.tif]

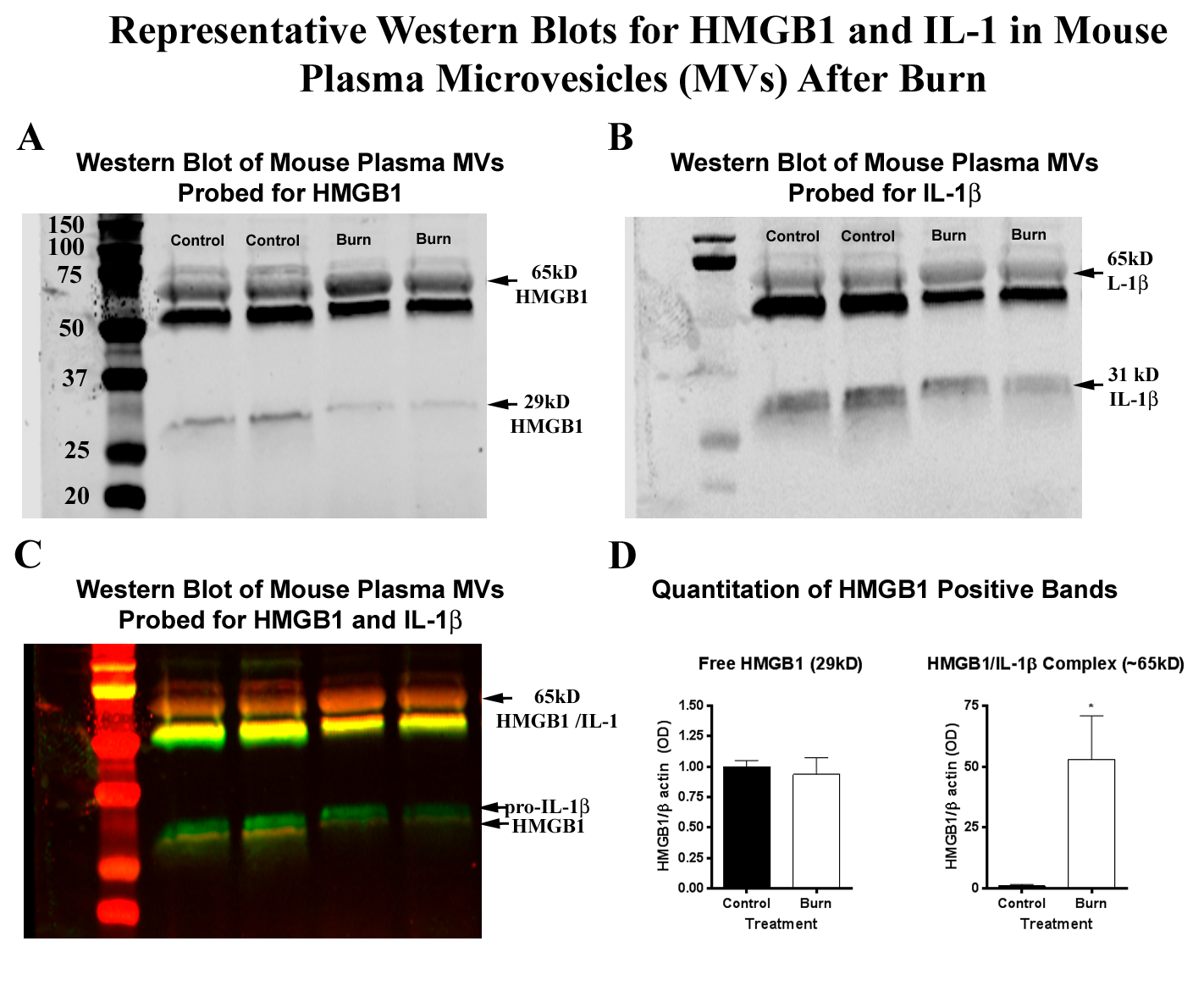

Supplement: S2 Fig — Microvesicles (MVs) were isolated by size using centrifugation (21,000g for 1 hour) and analyzed by Western Blot. (A) Probing for HMGB1 revealed three clear bands at 29kD (free HMGB1), approximately 55kD, and 65kD. (B) Probing for IL-1β revealed three clear bands at 31kD (pro-IL-1β), approximately 55kD, and 65kD. (C) Overlay of the staining for HMGB1 and IL-1β showed a separation of the 29 (free HMGB1) and 31kD (pro-IL-1β) bands but overlay of the 55kD and 65kD HMGB1 and IL-1β positive bands. (TIF) [file pone.0195335.s002.tif]

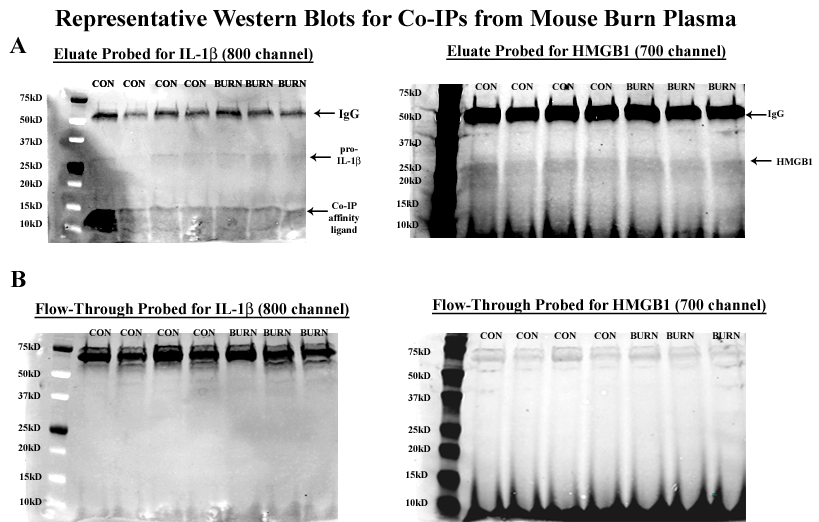

Supplement: S3 Fig — Co-immunoprecipitation was performed for HMGB1 and IL-1β in mouse control and burn plasma. Entire eluate and flow through for these samples are presented. (A) Entire blots of eluates probed for IL-1β and for HMGB1. Bands positive for IgG and pro-IL-1β as well as the Catch and Release® affinity ligand were observed. The blot probed for HMGB1 showed a broad band consistent with free HMGB1 near 29kD. (B) Entire western blots of flow through. Minimal IL-1β and HMGB1 were found in the flow through. (TIF) [file pone.0195335.s003.tif]

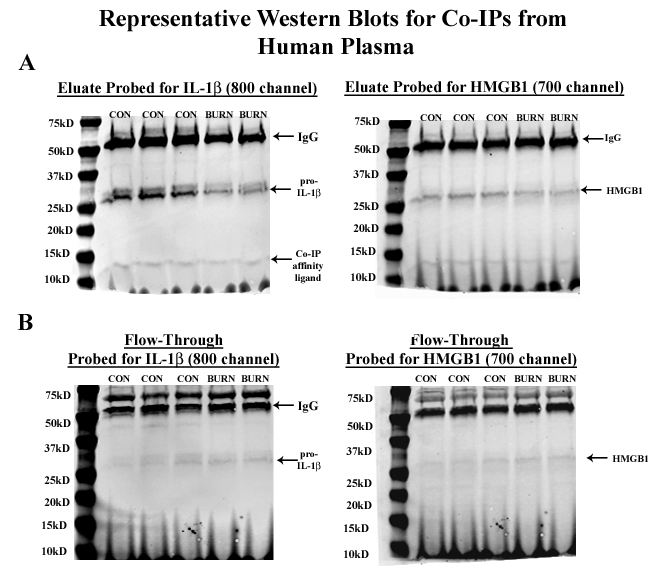

Supplement: S4 Fig — Co-immunoprecipitation was performed for HMGB1 and IL-1β in human control and burn plasma. Entire eluate and flow through for these samples are presented. (A) Entire blots of eluates probed for IL-1β and for HMGB1. Bands positive for IgG and pro-IL-1β as well as the Catch and Release® affinity ligand were observed. The blot probed for HMGB1 showed a broad band consistent with free HMGB1 near 29kD. (B) Entire western blots of flow through. Minimal pro-IL-1β and HMGB1 were found in the flow through. (TIF) [file pone.0195335.s004.tif]
